# A radiomics based method for prediction of prostate cancer Gleason score using enlarged region of interest

**DOI:** 10.1186/s12880-023-01167-3

**Published:** 2023-12-08

**Authors:** Haoming Zhuang, Aritrick Chatterjee, Xiaobing Fan, Shouliang Qi, Wei Qian, Dianning He

**Affiliations:** 1 College of Medicine and Biological Information Engineering, Northeastern University, Shenyang, China; 2 Department of Radiology, University of Chicago, 5841 S Maryland Ave, Chicago, IL 60637 USA

**Keywords:** Multiparametric MRI, Gleason score, Texture feature, Machine learning, Prostate cancer

## Abstract

**Background:**

Prostate cancer (PCa) is one of the most common cancers in men worldwide, and its timely diagnosis and treatment are becoming increasingly important. MRI is in increasing use to diagnose cancer and to distinguish between non-clinically significant and clinically significant PCa, leading to more precise diagnosis and treatment. The purpose of this study is to present a radiomics-based method for determining the Gleason score (GS) for PCa using tumour heterogeneity on multiparametric MRI (mp-MRI).

**Methods:**

Twenty-six patients with biopsy-proven PCa were included in this study. The quantitative T2 values, apparent diffusion coefficient (ADC) and signal enhancement rates (α) were calculated using multi-echo T2 images, diffusion-weighted imaging (DWI) and dynamic contrast-enhanced MRI (DCE-MRI), for the annotated region of interests (ROI). After texture feature analysis, ROI range expansion and feature filtering was performed. Then obtained data were put into support vector machine (SVM), K-Nearest Neighbor (KNN) and other classifiers for binary classification.

**Results:**

The highest classification accuracy was 73.96% for distinguishing between clinically significant (Gleason 3 + 4 and above) and non-significant cancers (Gleason 3 + 3) and 83.72% for distinguishing between Gleason 3 + 4 from Gleason 4 + 3 and above, which was achieved using initial ROIs drawn by the radiologists. The accuracy improved when using expanded ROIs to 80.67% using SVM and 88.42% using Bayesian classification for distinguishing between clinically significant and non-significant cancers and Gleason 3 + 4 from Gleason 4 + 3 and above, respectively.

**Conclusions:**

Our results indicate the research significance and value of this study for determining the GS for prostate cancer using the expansion of the ROI region.

## Background

Prostate cancer is among the most common cancers in men worldwide. The challenge in diagnosing prostate cancer (PCa) is not only to detect cancers but also to distinguish between non-clinically significant and clinically significant PCa. The current international standard for assessing the malignancy of prostate cancer is Gleason score (GS). GS is a staging method most commonly used in prostate cancer histology to assess aggressiveness with higher GS indicating more biologically aggressive cancer. In 2014, the International Society of Urological Pathology (ISUP) released supplementary guidance and an updated grading system for prostate cancer, termed the Grade Groups. This ISUP grading system is structured into five distinct grades: grade 1 (GS ≤ 3 + 3), grade 2 (GS 3 + 4), grade 3 (GS 4 + 3), grade 4 (GS 4 + 4, 3 + 5, 5 + 3) and grade 5 (GS 9–10). However, GS is determined using invasive procedures such as biopsy or after surgery and conventional MRI parameters haven’t been successful in determine the Gleason score. Recent AI (Artificial Intelligence) approach was shown some promise but better methods are needed [1]. The current diagnostic method for PCa is to achieve a histopathological diagnosis by puncture biopsy, which results in under-detection of clinically significant PCa and undegrading of cancer aggressiveness/Gleason score compared to prostatectomy [2]. Moreover, studies have shown that MRI is better at PCa diagnosis than transrectal ultrasound (TRUS) biopsies [3]. MRI is increasingly being used to diagnose prostate cancer. Multi-parametric MRI (mp-MRI) plays a pivotal role in the diagnosis of PCa by visualizing tumors within the prostate, increasing treatment options, and reducing unnecessary biopsies [4]. The core components of mp-MRI include T2-weighted imaging (T2W), diffusion-weighted imaging (DWI), and dynamic contrast-enhanced MRI (DCE-MRI), each of which provides distinct information [5]. Current diagnostic practice for mp-MRI follows the Prostate Imaging Reporting and Data System: Version 2.1 (PI-RADS v2.1) [6]. However, PI-RADS still has limited ability to detect and distinguish between non-clinically and clinically significant PCa, mainly due to the variability among readers [7].

Aggressiveness of PCa is associated with GS. It is important to distinguish the non-clinically significant cancers (Gleason 3 + 3, ISUP grade 1) from clinically significant cancers (Gleason 3 + 4 and above, ISUP grade ≥ 2), and to determine whether a patient should receive conservative treatment, such as active surveillance or local therapy, or surgical resection to improve survival and minimize the risk of missing the optimal treatment time or overtreatment. In addition, it is extremely important to differentiate between Gleason 3 + 4 (ISUP grade 2) and Gleason 4 + 3 and above (ISUP grade ≥ 3). In clinical significance, Gleason 3 + 4 (ISUP grade 2) represents a major differentiation score of 3, when the tumor is confined to the prostate gland, while Gleason 4 + 3 (ISUP grade 3) represents a major differentiation score of 4, which means that the tumor has traversed the peritoneum of the prostate gland and local lymph node metastasis and distant metastasis have occurred. Distinguishing between them therefore has a significant impact on treatment decisions and patient prognosis. Reese et al. found that these two scores have different biological aggressiveness with different proportions of tumors grades in both, leading to different biological behaviors of the tumor and prognosis of the patient [8]. However, as the lesions are very similar in terms of appearance and parametric features, it is difficult to distinguish between the two with the naked eye alone [9].

Radiomics using mp-MRI for PCa diagnosis is being actively investigated for lesion detection and classification. The lesion detection and classification approach typically analysis of the textural parameters of the lesion to assess its benignity and malignancy. Lopes et al. screened image features from DCE-MRI and DWI of prostate cancer for training and validation of texture features in the images to diagnose prostate cancer region size and Gleason score of prostate cancer [10]. Khalvati et al. designed a radiomics-based automated method for PCa detection using a support vector machine (SVM) model [11]. However, the recent PROSTATEx-2 Challenge highlighted that a lot of the work using radiomics hasn’t been able to achieve good results in predicting Gleason score for mp-MRI images, with only 2 out of 43 methods showing superiority to random guessing [1].

Common approaches to utilize mp-MRI in radiomics are to extract texture features in region of interest (ROI) and calculate texture parameters, and to assess the benignity or malignancy of the lesion based on the obtained parameters [12]. However, there may be textural features that are not useful or counterproductive to the assessment of benignity or malignancy, and since mp-MRI underestimates tumor volume, it is likely that the radiologist physician to outlines a small ROI compared to the actual tumor size [13]. These ROIs traced by radiologists based on MRI images are normally very conservative. The tumour volume delineated using MRI itself has shown to be highly underestimated when compared to the tumor volume on pathology slides [13]. Therefore, enlarged ROIs based on what the radiologists draw on MRI may be a better representation of the actual tumor on pathology to capture the tumor heterogeneity and to improve diagnosis using texture analysis. Therefore, we use machine learning and statistical methods for effective feature screening to obtain feature parameters that reflect tumor heterogeneity and lesion characteristics. The ROI was appropriately expanded to improve the accuracy of judging benign and malignant. In addition, most of the work has been done using biopsy as the reference standard. However, numerous studies have reported that there is undegrading of cancer aggressiveness/Gleason score on biopsy compared to prostatectomy [14–16]. Therefore, we used radical prostatectomy as the reference standard for training and validation of our methods.

Here, we presented an artificial intelligence-based method for determining the Gleason score for prostate cancer using tumour heterogeneity to predict the GS of PCa by imaging histology. We first trained and predicted for non-clinically significant (Gleason 3 + 3, ISUP grade 1) and clinically significant cancers (Gleason 3 + 4 and above, ISUP grade ≥ 2), followed by aggressiveness of clinically significant cancers (Gleason 3 + 4 versus Gleason 4 + 3 and above, ISUP grade 2 versus ISUP grade ≥ 3)). In addition, because the size of the ROI of mp-MRI is usually smaller than the actual lesion size on ground truth pathology, the expanded ROI area was used to get a better understanding of tumor heterogeneity and predict its GS more accurately.

## Methods

### Patients

This study involved a retrospective analysis of prospectively collected data, from 30 patients with known prostate cancer who underwent MRI before undergoing radical prostatectomy in hospital at the research center between February 2014 and February 2017. None of these patients had recently undergone radiotherapy or hormone replacement therapy, so there was no impact on MR images. The imaging tools and methods used varied between patients as their conditions were different from each other. As quantitative parameters such as apparent diffusion coefficient (ADC) and T2 values depended on imaging parameters such as b-values and TE used, the group with the highest number of patients using the same imaging modality was selected for this project [17].

### MR data

MRI data were acquired on a Philips Healthcare Achieva 3-T scanner with an endorectal coil. The endorectal coil was used, as it has better SNR and has been shown to improve cancer diagnosis, even though the current trend is to not use it due to cost and patient comfort [18]. Mp-MRI protocols included axial and coronal T2W, multi-echo T2-weighted images, DWI and DCE-MRI. MRI parameters were consistent in all patients. The MRI parameters used are described in detail in Table 1, which are compliant with PIRADS recommendations.

**Table 1:** MRI parameters

Imaging type	Pulse sequence	FOV (mm^2^)	Matrix size	In-Plane resolution (mm)	TR/TE (ms)	Slice thickness (mm)	Flip angle (°)
T2 map	TSE	160 × 160	210 × 210	0.75 × 0.75	7850/30–270	3	90
DWI	SE EPI	120 × 120	120 × 120	1.5 × 1.5	6093/80	3	90
DCE-MRI	T1-weighted FFE	250 × 385	200 × 308	1.25 × 1.25	4.8/3.3	3	10

*SE* spin echo, *TSE* turbo spin echo, *DCE* dynamic contrast-enhanced, *EPI* echo-planar imaging, *FFE* fast field echo

From the mp-MRI images, we calculated the DCE-MRI signal enhancement rates (α), ADC and T2 on a per-pixel basis. Current diagnostic practice using mp-MRI recommended the PI-RADS v2.1 [6], is conducted through T2W, DWI, and DCE-MRI. Prior studies have also demonstrated the effectiveness of T2 values, ADC, and α in distinguishing lesions [19]. Therefore, we selected these three quantitative parameters for the analysis.

The value of the ADC was calculated from DWI using Eq. 1.

1 $$S={S}_{0}\text{e}\text{x}\text{p}(-b\cdot ADC)$$

where b is the diffusion weighting factor, S_0_ is the un-diffused spin-echo signal, and S is the diffusion-weighted attenuated spin-echo signal. The b values of this experiment are 0, 50, 100, 150, 990, 1500 s/mm^2^.

The value of α was calculated from DCE-MRI with temporal resolution of 8.3 s using the empirical mathematical model according to Eq. 2 [19].

2 $$PSE\left(t\right)=A(1-{\text{e}}^{-\alpha t}){\text{e}}^{-\beta t}$$

where PSE is the percentage of signal enhancement, A is the amplitude of PSE, α is the rate of signal enhancement, and β is the elution rate. Α is chosen as it was found to have the highest diagnostic performance among the DCE-MRI parameters in our previous work [19].

Finally, the value of T2 was calculated from multi-echo SE imaging according to Eq. 3.

3 $$S={S}_{0}\text{e}\text{x}\text{p}(-{TE}/{T2})$$

where TE is the echo time, S is the signal strength corresponding to different echo times, and S_0_ is the signal strength at the moment when TE = 0. The TE of this experiment is from 30 ms to 270 ms with 30 ms interval.

The data in this study was processed using the same workstation for computing (Intel Core 5800 H processor, Nvidia 1060Ti graphics card, 8GB RAM), and the running environment was Matlab2019b (MathWorks, Natick, MA, USA).

### Histopathology and regions of interest delineation

The entire prostate gland was submerged in formalin after radical prostatectomy, then serially sectioned in a plane similar to the MR images. After being embedded in paraffin and stained with H&E, the whole prostate tissue sections were subsequently fixed on whole mount glass slides. An experienced pathologist (15 years of expertise) examined these tissue sections for prostate adenocarcinoma, and all malignant tumors were noted on the histology slides. The size of the case was based on the maximal extent of each lesion on the section, which was assigned a Gleason score and pathological stage. We identified all lesions with a maximal size greater than 5 mm that were included in the analysis after analyzing the tissue sections from the prostatectomy. For the purpose of to correlate the sections with the MR images, the sections were later imaged and digitalized using a bright field scanning microscope.

### ROI marking

**Fig. 1:**
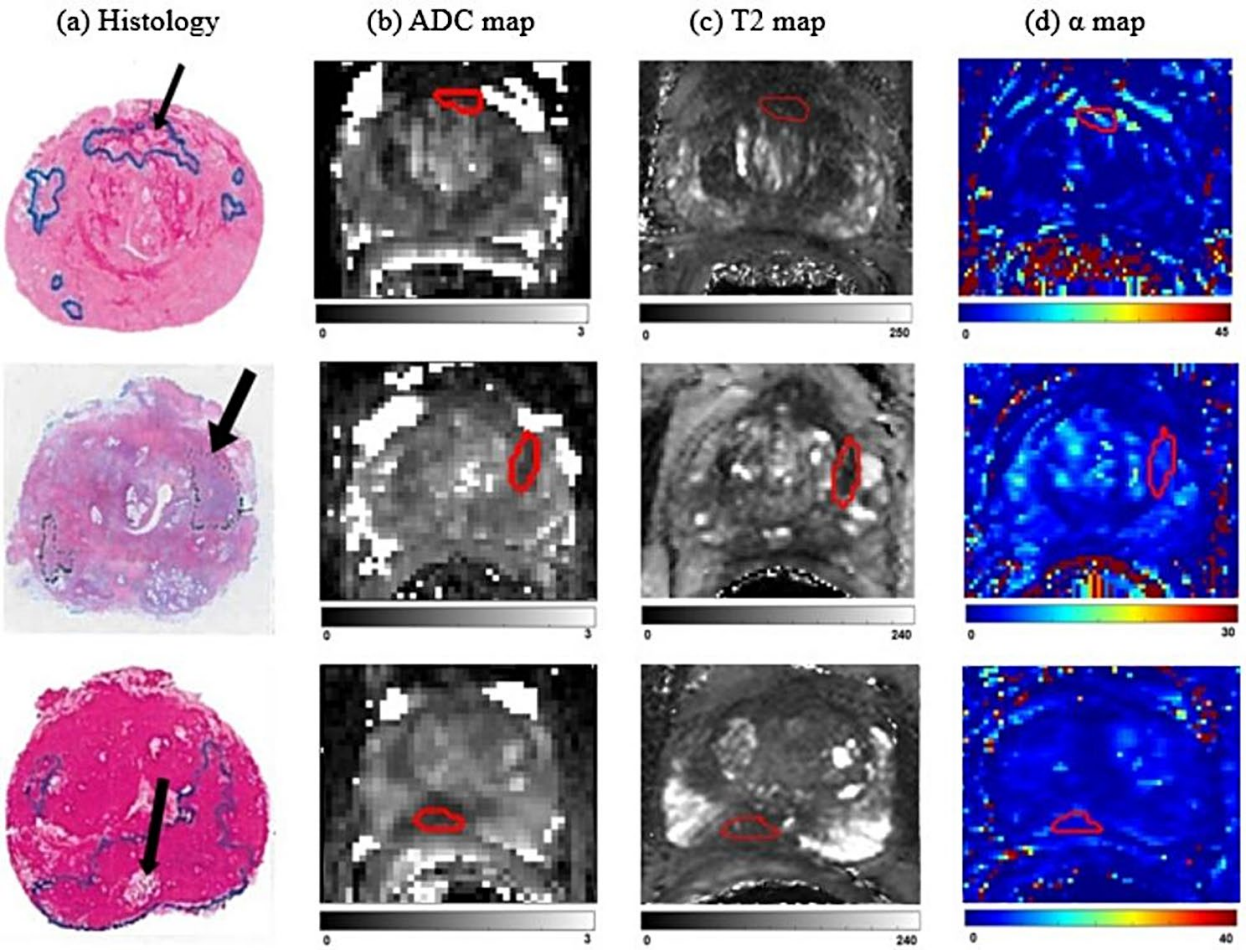
Three examples of images of the pathology, ADC(μm^2^ /ms), T2(ms) and α(%/s): **a** ROI of pathological image, **b** ROI of ADC image, **c** ROI of T2 image, **d** ROI of α image. The top, middle and bottom rows of the figure show the pathological results of Gleason 3 + 3 (ISUP grade 1), Gleason 3 + 4 (ISUP grade 2), and Gleason 4 + 3 (ISUP grade 3) respectively, and the predicted result after the method of this study are consistent with pathological results

The axial T2-weighted images were used as a standard to co-aligned images from mp-MRI sequences using rigid registration in the open-source medical imaging platform 3D Slicer and thus matched to the corresponding histological sections. The ROI of the cancer was drawn by the radiologist from registered whole mount histology and T2W imaging and applied to all calculated maps. The same shape and size were maintained using the 3D Slicer using the previously studied method [20]. Figure 1 shows three examples of (top to bottom row) images of the pathology, ADC, T2 and α of Gleason 3 + 3 (ISUP grade 1), Gleason 3 + 4 (ISUP grade 2), and Gleason 4 + 3 (ISUP grade 3) lesions with pathological finding.

### Texture feature extraction

Texture features were extracted from the gray level co-occurrence matrix (GLCM) of the ROI. The GLCM is defined by the joint probability density of pixels at two locations, which reflects not only the distribution characteristics of the greyscale levels, but also the location distribution characteristics between pixels of the same greyscale level. GLCM is the basis for calculating image texture features. Certain parameters are constructed based on its texture classification features, reflect the imaging characteristics of different prostate tissues. The four directions of 0°, 45°, 90° and 135° were chosen to generate the grey level co-occurrence matrix and six texture features were calculated, including Energy, Entropy, Contrast, Correlation, Homogeneity and Inverse Differential Moment (IDM). Due to the need for feature extraction of the entire prostate region in this study, the conclusions from this study can only be applied during the application process to imaging cases that are also consistent with MRI sequences covering the entire prostate region.

Their average in the four directions was then taken as the texture parameter for ROI. After calculating the ROI characteristic parameters of ADC, T2 and α respectively, the three maps of Energy, Entropy, Contrast, Correlation, Homogeneity and IDM are stored in a mat file along with the corresponding Gleason score for subsequent use.

Energy is a measure of the homogeneity of the distribution of grey levels in an image. When the heterogeneity of the tumour is low, the image will have a relatively homogeneous distribution of grey levels in local areas. Entropy is a measure of the randomness of the amount of information and the distribution of greyscales in an image. A higher entropy value in an image indicates a higher heterogeneity of the tumour. Contrast reflects a measure of the variation of greyscale in an image, with larger differences in local pixel greyscale values indicating higher heterogeneity of the tumour. Correlation is a parameter used to measure how similar the grey values in an image are in rows or columns. The smaller the correlation, the higher the heterogeneity of the image. Homogeneity reflects the distribution pattern of the image’s greyscale. The smaller the homogeneity, the higher the heterogeneity of the tumour. IDM reflects the variability of the textures on the image. The smaller the contrast, the higher the heterogeneity of the tumour [21].

### Expansion of ROI size

**Fig. 2:**
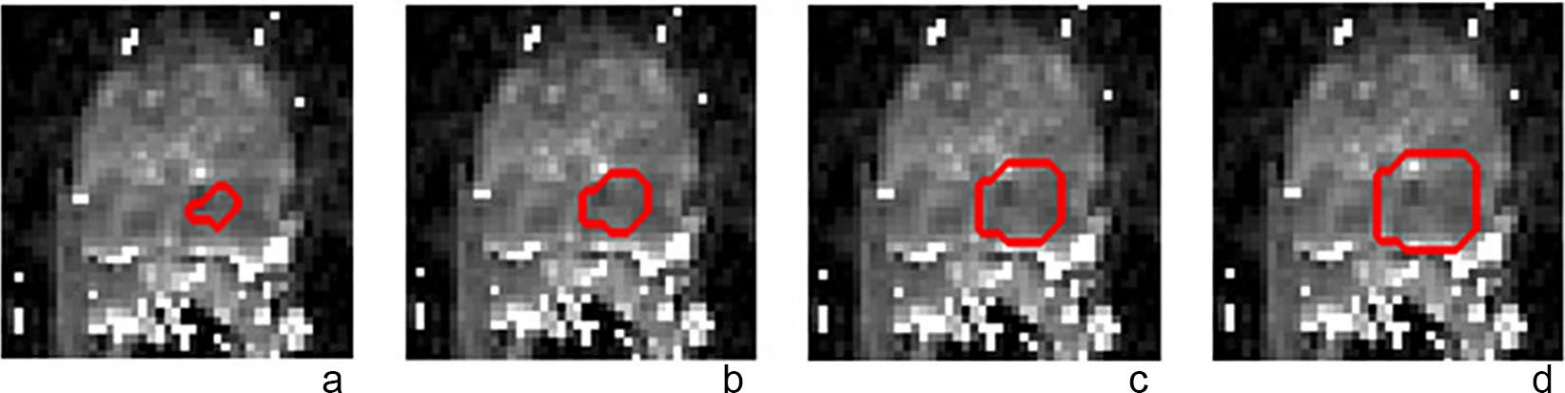
Original ROI and ROI after 1, 2 and 3 times inflated. **a**-**d**, ADC map with a GS of Gleason 3 + 4 (ISUP grade 2). The original ROI was predicted to be Gleason 3 + 3 (ISUP grade 1), the inflated once ROI was predicted to be Gleason 3 + 4 (ISUP grade 2), the inflated twice ROI was predicted to be Gleason 3 + 4 (ISUP grade 2) and the inflated 3 times ROI was predicted to be Gleason 3 + 3 (ISUP grade 1)

As radiologists usually mark the ROI on mp-MRI to a smaller extent than the actual lesion, an inflation operation is performed to expand the ROI size and thus obtain more accurately estimate of tumor heterogeneity and more feature parameters to test whether the accuracy could improve in subsequent classification results. The expansion was performed using square structural elements of 2 × 2 size. Although the radiologist marked ROI size is smaller than the actual lesion, the shape of the ROI is representation of the lesion outline. Therefore, when expanding the ROI in this experiment, only square structural elements are used, which is equivalent to proportionally expanding the ROI region without changing its original shape. The 2 × 2 size can be inflated by one pixel so that the best classification results are not lost due to the oversized structural elements. Each ROI is initially expanded by 1, 2 and 3 times respectively, before seeing if it needs to be expanded by 4 turns depending on the trend of the results. Figure 2 illustrates the ROI size changes: (a) the original ROI size, (b) the ROI size after 1 turn of expansion, (c) the ROI size after 2 turns of expansion, and (d) the ROI size after 3 turns of expansion.

### Radiomics analysis

First of all, feature selection was performed using the 21 texture features. In this study the ReliefF function is used for feature selection by the K-Nearest Neighbor (KNN) algorithm. Texture features are given different weights based on their ability to discriminate between close samples and their relevance. A weight > 0 indicates a strong influence on tumour heterogeneity, while a weight ≤ 0 indicates no influence or a negative influence on tumour heterogeneity. Parameters that had a small effect on tumour heterogeneity or a negative influence factor (i.e., no effect) were discarded, leaving only the feature parameters with a large influence factor.

Secondly, the LIBSVM toolbox (an effective, fast and easy to use package for SVM pattern recognition and regression), KNN function and Bayesian function were used for machine learning. The reason for choosing these three classifiers is: (i) they are very classical in the field of machine learning; (ii) previous studies on the classification of PCa GS by using machine learning used these classifiers and got good results [22]. These studies did not use mp-MRI but single type of MRI, so we want to use these three classifiers to classify GS by mp-MRI [23]. the optimal parameters were found in the LIBSVM parameter selection after experimenting with changing the values of C, G and T. C is the loss function, the main function is to set the parameters of C-SVC, E-SVR and V-SVR. It is C that determines the three different types of SVM applicable to a classification or regression problem. G is the setting of gamma function in the kernel function, mainly for polynomial G is the setting of the gamma function in the kernel function, mainly for the polynomial, rbf and sigmoid kernel functions, T is the type of kernel function, the types of kernel functions available are linear kernel function, polynomial kernel function, RBF kernel function and sigmoid kernel function, the best result in this experiment is the RBF kernel function. The KNN classifier was set to the number of nearest neighbors to 5 using “Euclidean” as the metric distance, and set the “nearest” rule to classify the samples. The Bayesian classifier is set up to model the data using a normal distribution and uses a priori probability and Cost of misclassification to help with classification.

In this study, the data was grouped and trained based on Gleason scores of 3 + 3, 3 + 4, and 4 + 3 (ISUP grade 1,2,3) and above. The triple classification was performed using SVM, KNN, and Bayesian. SVM uses a one-versus-one approach for classification, KNN triple classifies the data by counting the percentage of nearest neighboring categories, and Bayesian using normal distribution can also directly triple classify the data. These three classifiers are utilized for binary classification between clinical significance (Gleason 3 + 4 and above, ISUP grade ≥ 2) and non-clinical significance (Gleason 3 + 3, ISUP grade 1), and for binary classification between Gleason 3 + 4 (ISUP grade 2) and Gleason 4 + 3 and above (ISUP grade ≥ 3).

### Sample augmentation

Due to unbalanced the number of samples from different GSs in training and testing with machine learning, the synthetic minority oversampling technique (SMOTE) was used for sample improvement. SMOTE uses k-nearest neighbor algorithm to oversample the class that has a smaller number of samples in performing the classification processing so that both classes have the same number of samples. In the experiment, SMOTE was applied to the training set after a 5-fold cross-validation and separated from the test set to avoid data leakage.

### Statistics analysis

Statistical analysis was performed using Matlab2019b (MathWorks, Natick, MA, USA). First, we used an analysis of variance (ANOVA) to determine whether texture features in the GS triple classification had a significant effect on GS grouping (p < 0.05). After feature selection of texture features, we used a two-tailed t-test to calculate whether there was a significant feature between the screened texture features and the GS group (p < 0.05). Then, means and standard deviations were calculated for clinical significance (Gleason 3 + 4 and above, ISUP grade ≥ 2) and non-clinical significance (Gleason 3 + 3, ISUP grade 1), Gleason 3 + 4 (ISUP grade 2), and Gleason 4 + 3 and above (ISUP grade ≥ 3).

Considering the number of experimental samples, this experiment uses the number of training set: number of test set = 8:2, so the experiment is conducted using the 5-fold cross-validation method, in which the data are randomly divided into five subsets of equal size. Four subsets are taken as the training set and one subset as the test set each time, and the accuracy of predicting GS is reported as the average obtained across 5-folds, which can ensure the stability of the results and eliminate the influence of randomness.

## Results

### Patient characteristics

Twenty-seven of the 30 patients received imaging modalities including T2-weighted imaging, DWI with the same b-value, T2 mapping with the same TE and DCE-MRI, meeting the criteria for inclusion. However, one patent’s data was further excluded because his DCE-MRI sequence did not cover the entire prostate region. A total of 26 patients’ data were ultimately used for the trial. In these cases, according to the pathologist labeled, 13 Gleason 3 + 3 (ISUP grade 1); 29 Gleason 3 + 4 (ISUP grade 2); 7 Gleason 4 + 3 (ISUP grade 3); 1 Gleason 4 + 5 (ISUP grade 5) cancer lesion.

### Classification by texture feature parameters

**Fig. 3:**
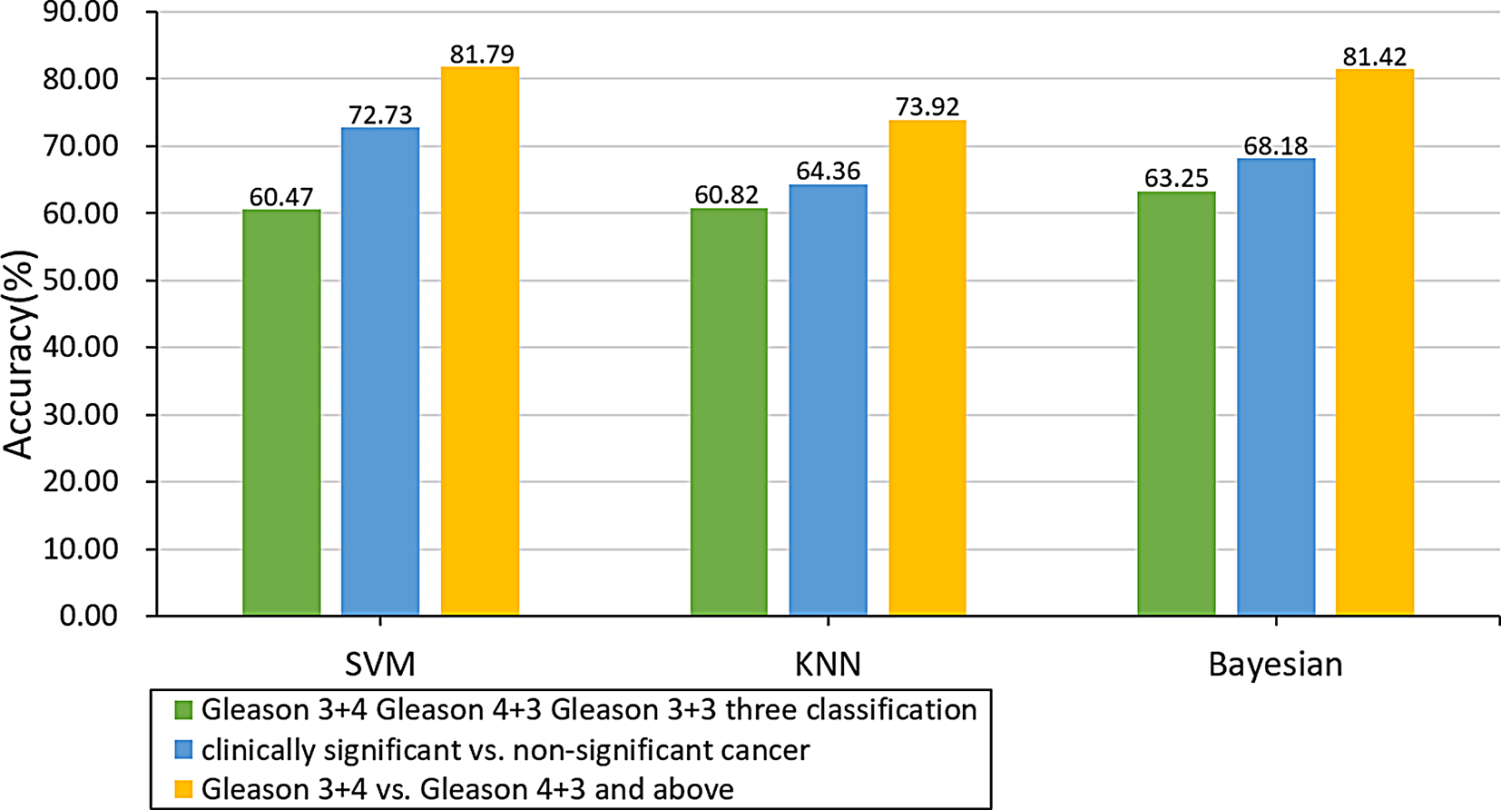
Accuracy of three methods for predicting different Gleason scores

Figure 3 shows the results of classify the ROIs with three different Gleason classifications: Gleason 3 + 3 (ISUP grade 1), Gleason 3 + 4 (ISUP grade 2), and Gleason 4 + 3 and above (ISUP grade ≥ 3), using three classifiers, KNN, SVM and Bayesian. There are moderate accuracy using the above texture parameters to bifurcate clinically significant (Gleason 3 + 4 and above, ISUP grade ≥ 3)) and non-clinically significant (Gleason 3 + 3, ISUP grade 1), Gleason 3 + 4 (ISUP grade 2) and Gleason 4 + 3 and above (ISUP grade ≥ 3) respectively by SVM, KNN and Bayesian.

We performed an analysis of variance (ANOVA) on the 21 texture features for performing the three classifications, and a total of eight features such as ADC, ALPHE, and ADC-homogeneity had a significant effect on the classification (p < 0.05). Consequently, we performed feature selection for 21 feature parameters, and the weight values of each texture feature can be found in Table 2.

**Table 2:** Texture feature weight values

Texture features	Clinically significant vs. non-significant cancer	Gleason 3 + 4 (ISUP grade 2) vs. Gleason 4 + 3 and above (ISUP grade ≥ 3)
ADC	**0.0237**	-0.0449
ALPHE	**0.0669**	-0.0090
T2	**0.0620**	**0.0231**
ADC-IDM	-0.0014	-0.0128
ADC-contrast	**0.0441**	**0.0632**
ADC-correlation	-0.0053	**0.1528**
ADC-energy	-0.0162	-0.0169
ADC-entropy	-0.0254	**0.0610**
ADC-homogeneity	**0.0195**	**0.0128**
DCE-IDM	-0.0157	-0.0770
DCE-contrast	-0.0069	-0.0474
DCE-correlation	**0.0377**	**0.0519**
DCE-energy	**0.0116**	**0.0150**
DCE-entropy	**0.0282**	-0.0450
DCE-homogeneity	**0.0037**	**0.0623**
T2-IDM	**0.0080**	-0.0388
T2-contrast	-0.0053	-0.0319
T2-correlation	-0.0156	-0.0622
T2-energy	**0.0067**	-0.0471
T2-entropy	**0.0413**	-0.0014
T2-homogeneity	**0.0307**	-0.0387

The value in bold means positive weight

Among the weight values of each texture feature, a negative weight value means that it is not related to the classification result of dichotomy, and a larger weight value means that it is more closely related to the classification result. We selected the factors that had a greater impact on the classification. Therefore, when classifying non-clinically significant and clinically significant, the selected characteristic parameters are ADC, ALPHE, T2, ADC-contrast, ADC-homogeneity, DCE-correlation, DCE-energy, DCE-entropy, DCE-homogeneity, T2-IDM, T2-energy, T2-entropy, T2-homogeneity, as texture features for binary classification. Their means, standard deviations and significant difference can be found in Table 3.

**Table 3:** Comparison of means, standard deviations and significant difference between clinically significant and non-significant cancer. The unit for ADC, T2, and α is μm^2^ /ms, ms, %/s, respectively.

Texture features	Mean	Standard deviation	*P* value
ADC	1.45/1.27	0.37/0.16	0.0103
ALPHE	7.43/7.91	0.71/0.51	0.0025
T2	104.21/114.41	25.81/24.11	0.0067
ADC-contrast	44.13/32.29	26.01/16.55	0.0262
ADC-homogeneity	41.12/32.99	15.31/13.77	0.0227
DCE-correlation	0.33/0.44	0.24/0.19	0.0120
DCE-energy	0.08/0.02	0.10/0.03	0.0035
DCE-entropy	0.29/0.41	0.15/0.09	0.0002
DCE-homogeneity	0.49/0.42	0.13/0.08	0.0122
T2-IDM	316.57/190.29	211.65/143.17	0.0047
T2-energy	0.03/0.01	0.03/0.01	0.0142
T2-entropy	0.51/0.55	0.11/0.07	0.0449
T2-homogeneity	0.55/0.47	0.08/0.04	0.0005

**Fig. 4:**
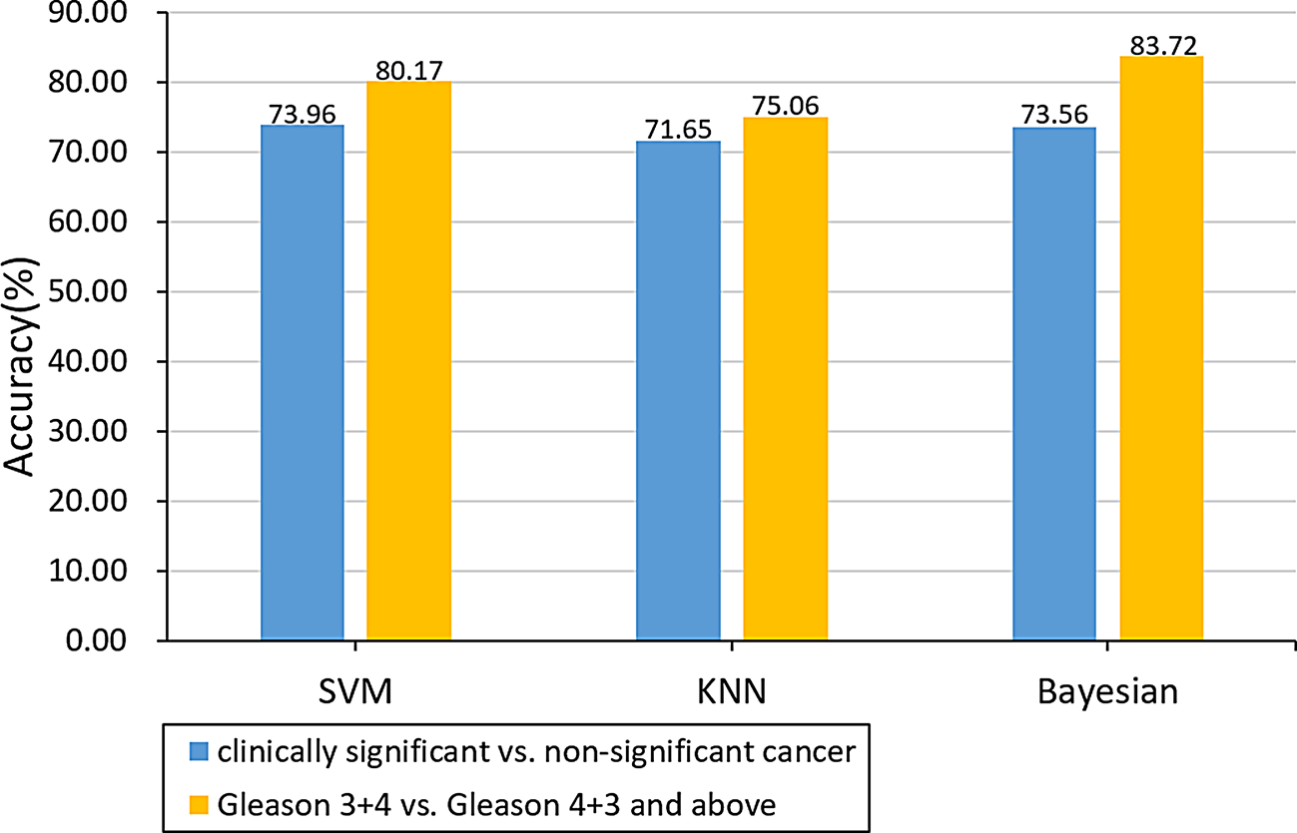
Comparison of classification accuracy after feature selection

When classifying Gleason 3 + 4 (ISUP grade 2) and Gleason 4 + 3 and above (ISUP grade ≥ 3), the selected feature parameters are T2, ADC-contrast, ADC-correlation, ADC-entropy, ADC-homogeneity, DCE-correlation, DCE-energy, DCE-homogeneity, as texture features for binary classification. Their means, standard deviations and significant difference can be found in Table 4. Figure 4 shows the new classification results. The accuracy results improved for all classifications, except for SVM to distinguish Gleason 3 + 4 (ISUP grade 2) from Gleason 4 + 3 and above (ISUP grade ≥ 3).

**Table 4:** Comparison of means, standard deviations and significant difference between Gleason 3 + 4 (ISUP grade 2) and Gleason 4 + 3 and above (ISUP grade ≥ 3)

Texture features	Mean	Standard deviation	*P* value
T2	89.96/109.36	14.54/26.19	0.0012
ADC-contrast	26.77/46.19	13.27/23.67	0.0020
ADC-correlation	0.55/0.41	0.02/0.14	0.0001
ADC-entropy	0.32/0.36	0.04/0.06	0.0041
ADC-homogeneity	0.49/0.46	0.05/0.06	0.0399
DCE-correlation	0.14/0.29	0.19/0.26	0.0127
DCE-energy	0.15/0.08	0.06/0.09	0.0037
DCE-homogeneity	0.59/0.52	0.08/0.13	0.0167

**Fig. 5:**
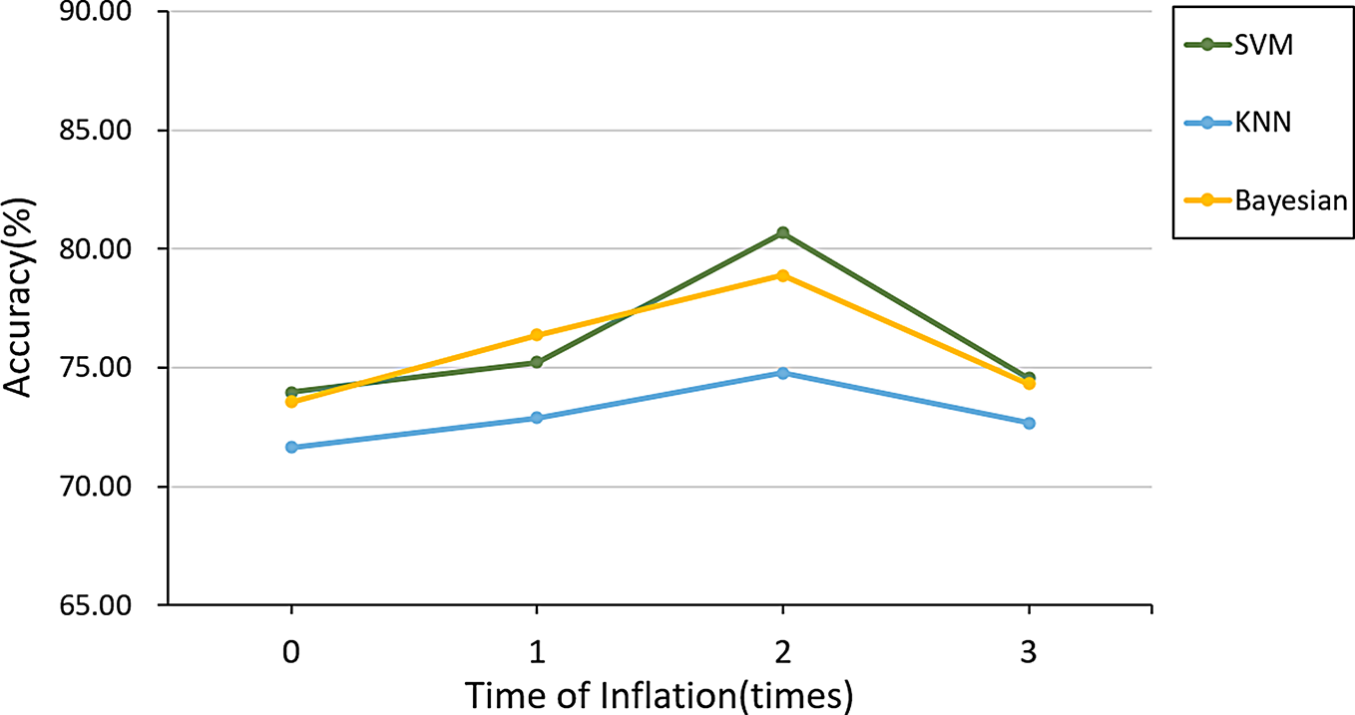
The classification accuracy of different times of inflation ROI for clinically significant and non-significant cancers.

The effect of ROI size on accuracy to distinguish between clinically significant and non-significant cancers was investigated by inflating all the ROIs 1, 2, and 3 times. The classification accuracy and the trend of its classification accuracy is shown in Fig. 5. The accuracy increased with increased ROI size (1 and 2 times, with 2 times having the highest accuracy) compared to original. ROI inflated 3 times did not show any improvements. Therefore, the highest accuracy was obtained with an accuracy of 80.67% by the SVM classifier when the ROI was inflated by 2 times.

**Fig. 6:**
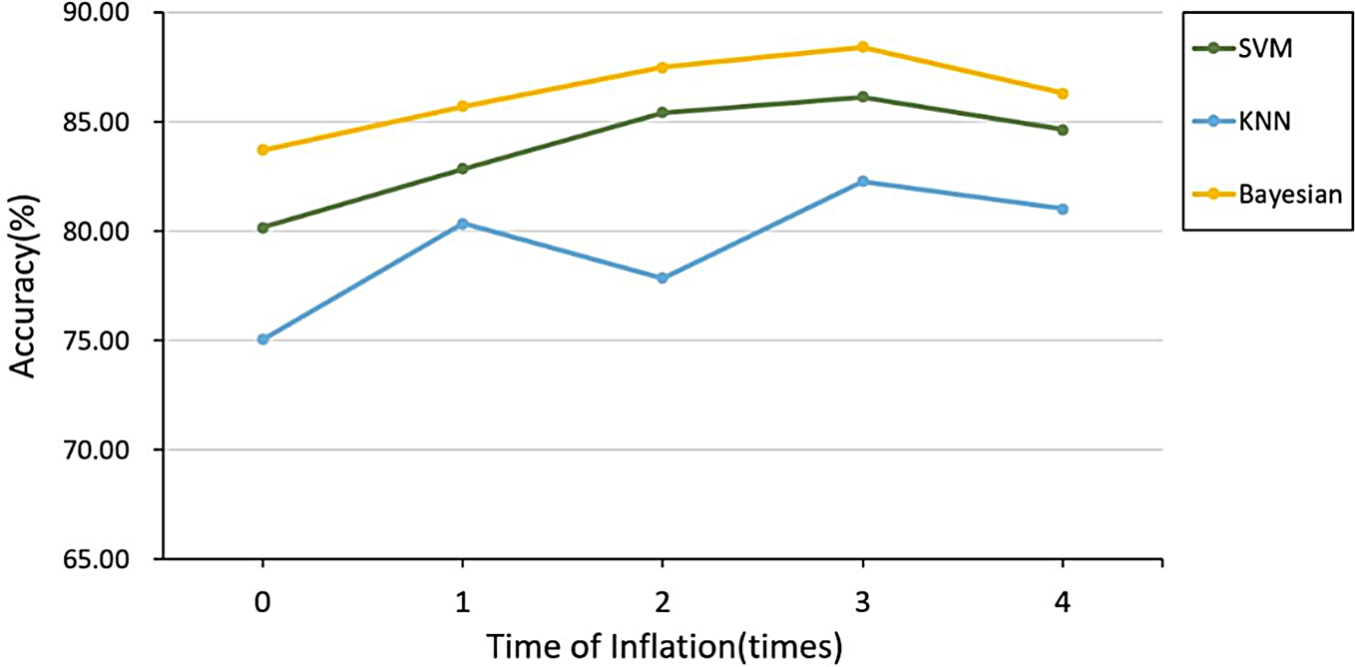
The classification accuracy of different times of inflation ROI for Gleason 3 + 4 (ISUP grade 2) and Gleason 4 + 3 and above (ISUP grade ≥ 3).

In the following, we tested the classification accuracy of ROI region size for Gleason 3 + 4 (ISUP grade 2) and Gleason 4 + 3 and above (ISUP grade ≥ 3). The results and the trend of its classification accuracy is shown in Fig. 6. The accuracy increased with increased ROI size (1, 2 and 3 times, with 3 times having the highest accuracy) compared to original. ROI inflated 4 times did not show any improvements. As a result, the highest accuracy of 88.42% was obtained by the Bayesian classifier at the 3rd time of inflation.

## Discussion

In this study, we proposed a radiomics-based method for prediction of prostate cancer GS using enlarged ROI. Binary classification of ROI using SVM, KNN and Bayesian classifier by virtue of texture feature analysis and feature selection. When applying the texture feature parameters for classification, both the feature selection and the method of expanding ROI can effectively improve the classification accuracy and obtain good results. The final classification accuracy for clinically significant (Gleason 3 + 4 and above, ISUP grade ≥ 2) and non-significant cancers (Gleason 3 + 3, ISUP grade 1) was 80.67%. The classification accuracy of Gleason 3 + 4 (ISUP grade 2) and Gleason 4 + 3 and above (ISUP grade ≥ 3) was 88.42%.

In the process of classifying non-clinically significant and clinically significant of prostate cancer, as well as Gleason 3 + 4 (ISUP grade 2) and Gleason 4 + 3 and above (ISUP grade ≥ 3), we used the relief function for feature selection, and the function used the KNN algorithm. In high-dimensional feature samples, some representative features are selected to reduce the sample feature dimension. It can be seen from Table 2 that among the weight values of various texture features, the negative weight value means that it has nothing to do with the classification result, and the numerical value of the weight value is positively correlated with the correlation of the classification result. After feature selection, by comparing the results in Fig. 4, it can be seen that the accuracy of the classification has improved more obviously, indicating that it is available to improve the accuracy by using feature parameters with greater influence and excluding some feature parameters with less or no influence in the classification process.

When radiologists outline cancer outline using mp-MRI, lesion ROI area is usually smaller than the lesion, which leads us to calculate the texture parameters according to the original ROI, thus the whole prostate cancer lesion area cannot be included and some texture features and information about the tumor heterogeneity are lost.

The sizes of ROIs had great impact on distinguishing between cancer non-clinically significant and clinically significant cancer. The accuracy of the classification showed a trend of increasing and then decreasing as inflating 1, 2, and 3 times original ROIs (Fig. 5). Therefore, the highest accuracy was obtained by the SVM classifier when the ROI was inflated by 2 times. There is a large improvement after doing feature selection only. In the process of Gleason 3 + 4 (ISUP grade 2) and Gleason 4 + 3 and above (ISUP grade ≥ 3) classification, the accuracy rate showed a rising trend as the ROI was inflated for 1, 2, and 3 times (Fig. 6). Therefore, we inflated the ROI of Gleason 3 + 4 (ISUP grade 2) and Gleason 4 + 3 and above (ISUP grade ≥ 3) for the 4th time. From the results, we can see that the accuracy of its classification decreases, so the highest accuracy was obtained by the Bayesian classifier at the 3rd time of inflation. Compared to previous studies, the current study demonstrated higher accuracy in classification [1, 24, 25]. Dikaios et al. calculated the accuracy of the Gleason score assessment of PCa by two radiologists with 5 years of experience in prostate mp-MRI, and the final assessment accuracy was 0.67 [24]. Chaddad et al. predicted GS by radiomics and the model achieved an average of the area under the curves of the receiver operating characteristic (ROC) of 83.40, 72.71, and 77.35% to predict GS groups (G1) = 6; 6 < (G2) < (3 + 4) and (G3) ≥ 4 + 3, respectively [25]. In a worldwide challenge - PROSTATEx-2 Challenge, only 2 teams showed any success as predicting Gleason score using AI on MRI data [1]. Thus, our approach to Gleason score determination of prostate cancer using tumor heterogeneity through radiomics can assist physicians in diagnosis and improve the accuracy of Gleason score. However, due to the small cohort it is difficult to determine whether this study is particularly beneficial for a specific clinical situation or patient subgroup. More studies are needed to determine this.

Our study has several limitations. First, the classification accuracy of non-clinically significant and clinically significant cancer is low compared with that of Gleason 3 + 4 (ISUP grade 2) and Gleason 4 + 3 and above (ISUP grade ≥ 3). The reason could be that the Gleason pattern of the major part of both tissues is 3, while only the secondary Gleason pattern is different. Therefore, it could not be able to discriminate well in terms of specificity such as texture features, and there are too few parameters of texture features with classification value, which leads to its classification accuracy being lower than that of Gleason 3 + 4 (ISUP grade 2) and Gleason 4 + 3 and higher (ISUP grade ≥ 3). Second, the accuracy is low when performing triple classification, which could be due to the fact that the small sample size of ROIs with Gleason 4 + 3 and higher (ISUP grade ≥ 3), and it cannot discriminate this triple classification well by machine learning. Moreover, due to the subjective interpretation of mp-MRI, large variability is seen among radiologists, and thus the classification results using this method may differ from radiologists’ judgments. Finally, this study is a feasibility study focused on radiomics analysis, so only patients with identical imaging protocols are included to minimize the impact of image acquisition parameters on the results. This led to a small sample size for this study, so a larger number of patient data is needed in subsequent research to validate the results and analyze the impact of different imaging protocols.

Future work should increase the number of patients and thus providing more data for the extraction of texture parameters. Furthermore, deep learning methods can be used to classify their GS using the expanded ROI [26].

## Conclusions

In this study, we proposed a method based on ROI expansion to determine the GS of prostate cancer using tumor heterogeneity. The segmented ROI was used for feature selection, texture feature analysis, and binary classification using SVM, KNN, and Bayesian classifiers. When applying the texture feature parameters for classification, both the feature selection and the method of expanding ROI can effectively improve the classification accuracy and obtain good results. Thus, the radiologist can choose to enlarge the ROI area accordingly to enhance the accuracy of the diagnosis. The final classification accuracy for clinically significant (Gleason 3 + 4 and above, ISUP grade ≥ 2) and non-significant cancers (Gleason 3 + 3, ISUP grade 1) was 80.67%. The classification accuracy of Gleason 3 + 4 (ISUP grade 2) and Gleason 4 + 3 and above (ISUP grade ≥ 3) was 88.42%. Therefore, urologists can choose the optimal treatment option based on the accurate classification of lesion aggressiveness, along with more precise lesion volume. It will help them decide between localized therapies such as prostate ablation for localized and less aggressive cancers, while radical treatment like prostatectomy and radiation for more aggressive cancers. The classification results indicate the research significance and value of this study on the determination of Gleason score for prostate cancer based on ROI expansion using tumor heterogeneity.

## Data Availability

Data are available upon request. Please contact corresponding author at hedn@bmie.neu.edu.cn.

## List of abbreviations

ISUP: International Society of Urological Pathology
GS: Gleason score
PCa: Prostate cancer
mp-MRI: Multiparametric MRI
ADC: Apparent diffusion coefficient
α: Signal enhancement rates
DWI: Diffusion-weighted imaging
DCE-MRI: Dynamic contrast-enhanced MRI
ROI: Region of interests
SVM: Support vector machine
KNN: K-Nearest Neighbor
T2W: T2-weighted imaging
PI-RADS: Prostate Imaging Reporting and Data System
GLCM: Gray level co-occurrence matrix
IDM: Inverse Differential Moment
ANOVA: Analysis of Variance
TRUS: Transrectal ultrasound
